# Perirenalfat thickness is associated with bone turnover markers and bone mineral density in postmenopausal women with type 2 diabetes mellitus

**DOI:** 10.3389/fendo.2022.990667

**Published:** 2022-10-25

**Authors:** Wei Wang, Rong Huang, Ping Tai Tang, Mei Tu, Xiu Li Guo

**Affiliations:** ^1^ Department of Endocrinology, Longyan First Affiliated Hospital of Fujian Medical University, Longyan, China; ^2^ Department of Radiology, Longyan First Affiliated Hospital of Fujian Medical University, Longyan, China

**Keywords:** bone turnover markers, bone mineral density, postmenopausal women, type 2 diabetes mellitus, perirenal fat thickness

## Abstract

**Objectives:**

Emerging evidence demonstrated that perirenal fat may modulate bone metabolism through several pathological pathways. This study was aimed to assess the associations between perirenal fat thickness (PrFT) and bone turnover markers (BTMs) and bone mineral density (BMD) in postmenopausal women with type 2 diabetes mellitus (T2DM) and further explore the correlation between PrFT and osteoporosis.

**Methods:**

In this cross-sectional study, a total of 626 participants with complete data were enrolled in this study. Demographic and anthropometric information was collected. Biochemical parameters and BTMs were determined. PrFT and BMD were measured by computed tomography and dual-energy x-ray absorptiometry, respectively. Correlation analysis and regression models were used to assess the associations between PrFT and BTMs and BMD. The multiple binomial logistic regression model was used to estimate the independent variables of PrFT for osteoporosis.

**Results:**

Overall, the prevalence of osteoporosis was 38.7%. PrFT was negatively correlated with β-cross-linked C-telopeptide of type I collagen (β-CTX) (*r* = -0.216,*<* 0.001), L1–L4 BMD (*r* = -0.351, *<* 0.001), and T-score (*r* = -0.396, *<* 0.001). PrFT also remained significantly correlated with β-CTX (*β* = -0.291, *P<* 0.001), L1–L4 BMD (*β* = -0.109, *P=* 0.027), and L1–L4 T-score (*β* = -0.149, *P=* 0.001) after adjustment for other confounding factors. Furthermore, PrFT was also independently associated with osteoporosis after adjustment for other confounding factors; the OR (95% CI) was 1.13 (1.04–1.23). PrFT also seems to have a relatively good identifying value for osteoporosis. The area under the curve (AUC) value of PrFT in identifying osteoporosis was 0.766 (95% CI: 0.705–0.826, *P* < 0.001). The optimal cutoff value of PrFT was 15.2 mm (sensitivity: 72.5%, specificity: 79.8%).

**Conclusions:**

PrFT was significantly associated with β-CTX, BMD, and osteoporosis. These findings indicate that perirenal fat may play an important role in bone metabolism.

**Clinical Trial Registration:**

http://www.chictr.org.cn/, identifier (ChiCTR2100052032).

## Introduction

Type 2 diabetes mellitus (T2DM) and osteoporosis-related fractures have become major public health concerns in increasingly aging and obese populations. Emerging evidence from meta-analyses has reported an increase of fracture risk in T2DM compared with the healthy population (1, 2). The Study of Osteoporotic Fractures has also demonstrated that a history of T2DM is the strongest independent risk factor of low-energy subtrochanteric and diaphyseal fractures (3). T2DM linked with bone metabolism through these underlying pathophysiological mechanisms, including hyperglycemia, oxidative stress, and accumulation of advanced glycation end products caused by T2DM, which can compromise collagen properties, increases marrow adiposity, releases adipokines and inflammatory factors from visceral fat, and alters the function of osteocytes (4–6). Among the mechanisms proposed to explain the association between T2DM and bone metabolism, the abnormal accumulation of visceral fat can increase the secretion of adipokines and inflammatory factors such as leptin, tumor necrosis factor-α (TNF-α), and interleukin-6 (IL-6) (7, 8), which may increase bone resorption and decrease bone mineral density (BMD) (9, 10).

Visceral adipose tissue is well recognized as a type of “ectopic fat” that has adverse influences on bone metabolism, which can break the balance between bone formation and resorption. Clinical studies observed that increased visceral fat area (VFA) is strongly associated with decreased BMD and compromised bone structure (11, 12), whereas this relationship may vary with age and gender. Perirenal fat is a kind of measurable visceral adipose tissue located in the retroperitoneal space and enclosed from the inner side of the abdominal musculature to the surface of the kidney. Anatomical studies proved that perirenal fat has a complete vascular supply and lymphatic system (13). The anatomical structure and location of perirenal fat determined its specific biological characteristics, and it may modulate bone and energy metabolism through neural reflexes, adipokine secretion, and adipocyte interactions (14, 15). Despite the abundance of studies that have reported the effects of increased visceral fat mass accumulation on fracture risk and evaluated possible mechanisms on bone metabolism, the relationship between perirenal fat and bone metabolism is still uncertain. To put more insight into the potential effects of perirenal fat on bone metabolism, in this cross-sectional study, we mainly aimed to assess the association between perirenal fat thickness (PrFT) and bone turnover markers (BTMs) and BMD in postmenopausal women with T2DM and further explore the correlation between PrFT and osteoporosis.

## Materials and methods

### Study design

This cross-sectional study has consecutively enrolled individuals from the Department of Endocrinology at the Longyan First Affiliated Hospital of Fujian Medical University who fulfilled the study criteria between January 2022 and March 2022. All procedures were conducted in compliance with the Declaration of Helsinki. This study was approved by the Ethical Committee of Longyan First Affiliated Hospital of Fujian Medical University (LY-2020–069) and registered in Clinical Trials.Gov (ChiCTR2100052032). All participants enrolled in the study provided informed consent.

### Study population

**Figure 1:**
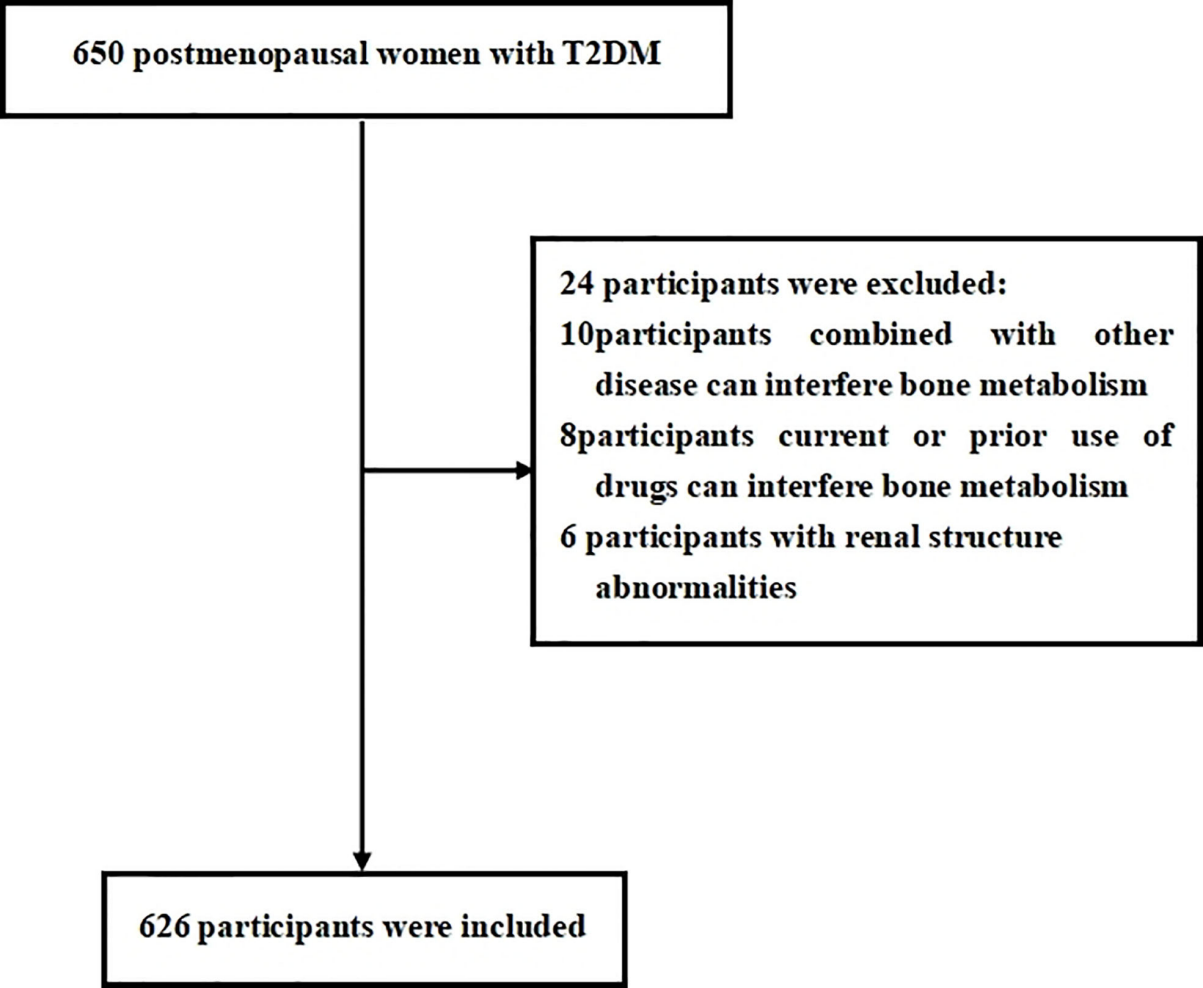
Flow diagram of the participants excluded and included in this study.

Eligibility study inclusion criteria were as follows: 1) diagnosed as having T2DM using the World Health Organization (2019) criteria; 2) postmenopausal women with 12 consecutive months of amenorrhea. Participants were excluded if they have the following: 1) a history of chronic diseases that can interfere with bone metabolism (i.e., renal, hepatic, cardiac, thyroid, and rheumatic diseases); 2) current or prior use of drugs that can interfere with bone metabolism (i.e., glucocorticoids, antiresorptive drugs, hormonal replacement therapy, calcium or vitamin D supplementation, antiosteoporosis therapy, thiazolidinediones, urate-lowering therapy); 3) renal structure abnormalities (tumors, cysts) or a history of renal region surgery. We estimated the sample size according to the requirement of multiple binomial logistic regression model. In this study, 12–15 variables may be put into the logistic regression model according to the principle of 5–10 events per variable, and the prevalence of osteoporosis is about 30%–40% in postmenopausal women with T2DM. Thus, we planned a sampling size of 500–600 participants. Overall, a total of 650 participants were screened. Among them, 626 participants meeting the inclusion and exclusion criteria were enrolled in this study. The flow diagram of excluded and included participants was presented in **Figure 1**.

### Anthropometric and laboratory assessments

The clinical data were collected by trained interviewers through a standard questionnaire, with each participant asked questions regarding her age, duration of diabetes, history of diseases that can interfere with bone metabolism and renal structure, current or prior use of drugs, smoking, drinking, and menopausal status. Participants who have any waking behaviors characterized by an energy expenditure ≤1.5 metabolic equivalents (e.g., watching television, reading, or reclining) were considered to have a sedentary behavior (16). Participants who smoke more than 4 cigarettes a week for at least 6 months continually or accumulatively were considered smoking. Participants who drink alcohol more than once a year were considered drinking. Information was also obtained by a review of medical records and laboratory data. Height and weight were measured to the nearest 0.1 cm and 0.1 kg, respectively. Subjects were wearing hospital gowns and had bare feet. Body mass index (BMI) was calculated as the weight divided by the square of height (kg/m^2^).

Laboratory assessments were conducted by standard methods using fasting venous blood samples, which were taken between 8:00 and 9:00 a.m. after fasting overnight for at least 12 h. High-density lipoprotein cholesterol (HDL-c), low-density lipoprotein cholesterol (LDL-c), triglycerides (TGs), uric acid (UA), phosphorus, and calcium were measured by an auto-biochemical analyzer (Roche Diagnostics Corporation). Glycosylated hemoglobin (HbA1c) was evaluated by high-performance liquid chromatography (HPLC) with a D10 set (Bio-Rad). BTMs such as osteocalcin (OC), β-cross-linked C-telopeptide of type I collagen (β-CTX), intact parathyroid hormone (iPTH), and 25-hydroxyvitamin D (25-OH-D) were measured by electro-chemiluminescence immunoassay method (Roche Diagnostics GmbH, Germany). Moreover, serum Thyroid stimulating hormone (TSH) was also measured to screen common thyroid diseases often accompanied by T2DM that can interfere with bone metabolism.

### Measurement of bone mineral destiny

Areal BMD was measured for the lumbar spine (L1–L4), total hip, and femoral neck by using Hologic Discovery Wi (Hologic, Marlborough, MA, USA) in a supine position. T-score was calculated according to Hologic densitometry reference value. The densitometry scan was performed by experienced radiographers who were blinded to clinical information. Longitudinal quality control check was performed daily using whole-body and L1–L4 lumbar spine phantom provided by the manufacturer. Cross-calibration was performed weekly to monitor variations between the systems. The precision error was 1.0% for the BMD measurement. Postmenopausal women with lumbar spine (L1–L4) or total hip or femoral neck T-score ≤2.5 or a history of fragility fracture were considered to have osteoporosis.

### Measurement of perirenal fat thickness

Revolution VCT 128 (General Electric, Milwaukee, WI, USA) was used to measure PrFT in a supine position for all participants. The scanning area was covered from the pubic symphysis and the 10th thoracic vertebra. Two radiologists were involved in the measurements of PrFT to reduce interoperator variability. Images were reconstructed with Advantage Windows 4.4 software (GE, Milwaukee, WI, USA) to obtain 1.25-mm-thick consecutive slices. Density [Hounsfield units (HU)] was used to differentiate perirenal fat from other tissues (window center: -100 HU; window width: 50–200 HU). The average of the maximal distance between the posterior wall of the kidney and the inner limit of the abdominal wall across the renal venous plane on both kidneys was defined as the PrFT (17). The interoperator agreement between the two radiologists is 0.91.

### Statistical analysis

Data were analyzed using SPSS 23.0 software (SPSS Inc. IBM, New York, USA). Descriptive data are expressed as means ± standard deviation (SD). Discrete variables were summarized in frequency tables (N, %). Statistical differences among groups were determined using one-way analysis of variance (ANOVA) followed by Tukey test for multiple comparisons. The chi-square (χ^2^) test or Fisher exact test was used for comparison of categorical variables. The relationship between PrFT and clinical variables was assessed by Pearson or Spearman correlation analysis. Multiple regression analysis was used to estimate independent associations between PrFT and BTMs and BMD after adjusting for potential confounding factors. The multiple binomial logistic regression model was used to estimate the independent variables of PrFT for osteoporosis after adjustment for other confounding factors. The receiver operating characteristic (ROC) curve was used to assess the identifying value of PrFT for osteoporosis. The optimal cutoff value was based on the greatest value of the Youden index. A two-tailed value of *P* < 0.05 was considered statistically significant.

## Results

Overall, a total of 626 participants with complete and available data were included in the final analysis. Characteristics of the overall study population based on tertiles of PrFT were summarized in **Table 1**. The mean age was 56.4 ± 6.1 years, diabetes duration was 6.9 ± 2.6 years, and mean BMI was 24.2 ± 2.8 kg/m^2^. There were no significant differences in age, diabetes duration, BMI, LDL-c, creatinine, and percentage of participants with hypertension, sedentary behavior, smoking, drinking, insulin therapy, and number of other hypoglycemic agents among the three groups. Increasing trends were observed in UA and TG across the PrFT tertiles (*<* 0.05). Decreasing trends were also observed in HDL-c across the PrFT tertiles (*P<* 0.05).

**Table 1:** Clinical and laboratory characteristics of participants based on tertiles of PrFT.

Variable	Total	T1 (<11.3mm)	T2 (11.3~15.5mm)	T3 (>15.5mm)	*P*
Age (years)	56.4 ± 6.1	56.3 ± 5.2	56.4 ± 6.0	56.5 ± 6.7	0.682
BMI (kg/m^2^)	24.2 ± 2.8	23.9 ± 2.8	24.5 ± 2.7	24.5 ± 3.0	0.186
Duration (years)	6.9 ± 2.6	6.8 ± 2.1	7.0 ± 2.7	7.1 ± 2.5	0.563
HbA1c (%)	8.8 ± 1.1	8.7 ± 1.0	8.9 ± 1.3	8.8 ± 1.1	0.358
TG (mmol/L)	2.24 ± 1.30	2.05 ± 1.33^ab^	2.19 ± 1.21^ac^	2.47 ± 1.34^bc^	0.009
HDL-c (mmol/L)	1.10 ± 0.23	1.14 ± 0.24^ab^	1.09 ± 0.23^ac^	1.05 ± 0.21^bc^	0.018
LDL-c (mmol/L)	3.56 ± 0.98	3.58 ± 0.95	3.60 ± 1.06	3.52 ± 0.92	0.679
UA (umol/L)	354.7 ± 87.9	339.6 ± 94.2^ab^	354.1 ± 85.4^ac^	370.4 ± 82.1^bc^	0.011
Creatinine (umol/L)	68.2 ± 13.4	66.2 ± 14.2	68.8 ± 12.6	67.0 ± 15.4	0.437
Hypertension, n (%)	240 (38.3)	70 (33.7)^b^	80 (38.1)	90 (43.2)^b^	0.130
Smoking, n (%)	15 (2.4)	4 (1.9)	5 (2.4)	6 (2.6)	0.814
Drinking, n (%)	97 (15.4)	30 (14.4)	35 (16.7)	32 (15.2)	0.813
Sedentary behavior, n (%)	256 (40.9)	76 (36.5)	90 (42.9)	90 (43.3)	0.293
Insulin, n (%)	66 (10.5)	20 (9.6)	24 (11.5)	22 (10.5)	0.815
Number of other hypoglycemic agents					
0	32 (5.1)	9 (4.3)	11 (5.3)	12 (5.7)	0.805
1	188 (30)	66 (31.7)	59 (28.3)	63 (30.0)	0.756
2	294 (47)	94 (45.2)	104 (50.0)	96 (45.7)	0.559
3	83 (13.5)	30 (14.4)	24 (13.3)	29 (11.7)	0.604

PrFT, perirenal fat thickness; BMI, body mass index; HbA1c, glycated hemoglobin; UA, uric acid; TG, triglyceride; HDL-c, high-density lipoprotein cholesterol; LDL-c, low-density lipoprotein cholesterol.

a P < 0.05: T1 vs. T2.

b P < 0.05: T1 vs. T3.

c P < 0.05: T2 vs. T3.

BTMs and BMD of participants based on tertiles of PrFT were presented in **Table 2**. The prevalence of osteoporosis was 38.7% in postmenopausal women with T2DM. The mean BMD was 0.87 ± 0.11 at the lumbar spine, 0.83 ± 0.11 at the total hip, and 0.73 ± 0.22 at the femoral neck. As expected, there were significant differences in the level of OC, β-CTX, L1–L4 BMD, and L1–L4 T-score among the three groups (*P<* 0.05). Decreasing trends were also observed in β-CTX, L1–L4 BMD, and T-score across the PrFT tertiles. Moreover, participants in the highest PrFT group have higher levels of OC than those of the other two groups.

**Table 2:** BTMs and BMD of participants based on tertiles of PrFT.

Variable	Total	T1 (<11.3mm)	T2 (11.3~15.5mm)	T3 (>15.5mm)	*P*
OC (ng/ml)	14.72 ± 6.74	14.11 ± 6.60^b^	14.04 ± 6.64^c^	16.07 ± 6.85^bc^	0.032
β-CTX (ng/ml)	0.47 ± 0.16	0.54 ± 0.18^ab^	0.47 ± 0.11^ac^	0.41 ± 0.16^bc^	< 0.001
25-OH-D (nmol/L)	67.4 ± 13.1	67.6 ± 12.5	66.6 ± 13.2	67.4 ± 13.1	0.678
iPTH (ng/L)	35.2 ± 13.2	33.7 ± 12.4	36.8 ± 14.2	34.9 ± 12.5	0.118
Calcium (mmol/L)	2.31 ± 0.11	2.32 ± 0.12	2.31 ± 0.10	2.31 ± 0.11	0.809
Phosphorus (mmol/L)	1.22 ± 0.18	1.22 ± 0.17	1.22 ± 0.17	1.23 ± 0.19	0.763
L1–L4 BMD (g/cm^3^)	0.87 ± 0.11	0.91 ± 0.12^ab^	0.87 ± 0.08^ac^	0.83 ± 0.11^bc^	< 0.001
L1–L4 T-score	-1.92 ± 0.87	-1.50 ± 1.04^ab^	-1.94 ± 0.69^ac^	-2.31 ± 0.59^bc^	< 0.001
Hip BMD (g/cm^3^)	0.83 ± 0.11	0.83 ± 0.09	0.83 ± 0.10	0.82 ± 0.12	0.462
Hip T-score	-0.92 ± 0.58	-0.90 ± 0.52	-0.92 ± 0.64	-0.94 ± 0.69	0.478
Femoral neck BMD (g/cm^3^)	0.73 ± 0.22	0.74 ± 0.18	0.73 ± 0.24	0.73 ± 0.26	0.376
Femoral neck T-score	-1.12 ± 0.68	-1.10 ± 0.78	-1.13 ± 0.58	-1.13 ± 0.64	0.488
Osteoporosis, n (%)	242 (38.7)	50 (24.0)^b^	67 (31.9)^c^	125 (60.1)^bc^	< 0.001

PrFT, perirenal fat thickness; OC, osteocalcin; β-CTX, β-cross-linked C-telopeptide of type I collagen; 25-OH-D, 25-hydroxyvitamin D; iPTH, intact parathyroid hormone; BTMs, bone turnover markers; BMD, bone mineral density.

^a^P < 0.05: T1 vs. T2.

^b^P < 0.05: T1 vs. T3.

^c^P < 0.05: T2 vs. T3.

The correlations between PrFT and BTMs and BMD in postmenopausal women with T2DM were presented in **Table 3**. The results showed that PrFT was negatively correlated with β-CTX (*r* = -0.216, *P><* 0.001), L1–L4 BMD (*r* = -0.351, *P<* 0.001), and L1–L4 T-score (*r* = -0.396, *P<* 0.001), whereas no correlations were found between PrFT and OC, 25-OH-D, iPTH, calcium, phosphorus, hip BMD and T-score, and femoral neck BMD and T-score. We also performed multiple linear regression analysis to determine independent variables of PrFT for β-CTX, L1–L4 BMD, and T-score. As shown in **Table 4**, the results showed that PrFT was negatively correlated with β-CTX (*β* = -0.252, *P<* 0.001), L1–L4 BMD (*β* = -0.309, *P<* 0.001), and L1–L4 T-score (*β* = -0.366, *P<* 0.001) after adjustment for clinical variables such as age, diabetes duration, BMI, TG, HDL-c, LDL-c, creatinine, UA, hypertension, sedentary behavior, smoking, and drinking (Model 1). Furthermore, PrFT remained significantly correlated with β-CTX (*β* = -0.291, *P<* 0.001), L1–L4 BMD (*β* = -0.109, *P>=* 0.027), and L1–L4 T-score (*β* = -0.149, *P=* 0.001) after additional adjustment for BTMs such as OC, 25-OH-D, iPTH, phosphorus, calcium (Model 2), and β-CTX (Model 3).

**Table 3:** Correlations between PrFT and BTMs and BMD in postmenopausal women with T2DM.

Variable	R	*P*
OC(ng/ml)	0.081	0.151
β-CTX(ng/ml)	-0.216	<0.001
25-OH-D(nmol/L)	-0.009	0.878
iPTH(ng/L)	0.103	0.092
Calcium(mmol/L)	-0.006	0.913
Phosphorus(mmol/L)	0.031	0.383
L1–L4 BMD(g/cm^3^)	-0.351	<0.001
L1–L4 T-score	-0.396	<0.001
Hip BMD(g/cm^3^)	-0.048	0.278
Hip T-score	-0.054	0.202
Femoral neck BMD(g/cm^3^)	-0.068	0.187
Femoral neck T-score	-0.086	0.134

PrFT, perirenal fat thickness; T2DM, type 2 diabetes mellitus; OC, osteocalcin; β-CTX, β-cross-linked C-telopeptide of type I collagen; 25-OH-D, 25-hydroxyvitamin D; iPTH, intact parathyroid hormone; BTMs, bone turnover markers; BMD, bone mineral density.

**Table 4:** Multivariate linear regression analysis of associations between PrFT and β-CTX, L1–L4 BMD, and L1–L4 T-score in postmenopausal women with T2DM.

Models	Unstandardizedcoefficient (B)	Standardizedcoefficient (β)	T	*P*
β-CTX				
Model 1	-0.010	-0.252	-4.346	< 0.001
Model 2	-0.012	-0.291	-5.202	< 0.001
L1–L4 BMD				
Model 1	-0.009	-0.309	-5.702	< 0.001
Model 3	-0.003	-0.109	-2.229	0.027
L1–L4 T-score				
Model 1	-0.088	-0.366	-7.006	< 0.001
Model 3	-0.039	-0.149	-3.280	0.001

Model 1: Adjusted for age, diabetes duration, HbA1c, BMI, TG, HDL-c, LDL-c, creatinine, UA, hypertension, sedentary behavior, smoking, and drinking. Model 2: Additional adjustment for bone turnover markers such as OC, 25-OH-D, iPTH, alkaline phosphatase (ALP) , calcium, and phosphorus based on Model 1. Model 3: Additional adjustment for β-CTX based on Model 2.

PrFT, perirenal fat thickness; T2DM, type 2 diabetes mellitus; BMI, body mass index; OC, osteocalcin; β-CTX, β-cross-linked C-telopeptide of type I collagen; 25-OH-D, 25-hydroxyvitamin D; iPTH, intact parathyroid hormone; HbA1c, glycated hemoglobin; UA, uric acid; TG, triglyceride; HDL-c, high-density lipoprotein cholesterol; LDL-c, low-density lipoprotein cholesterol.

**Figure 2:**
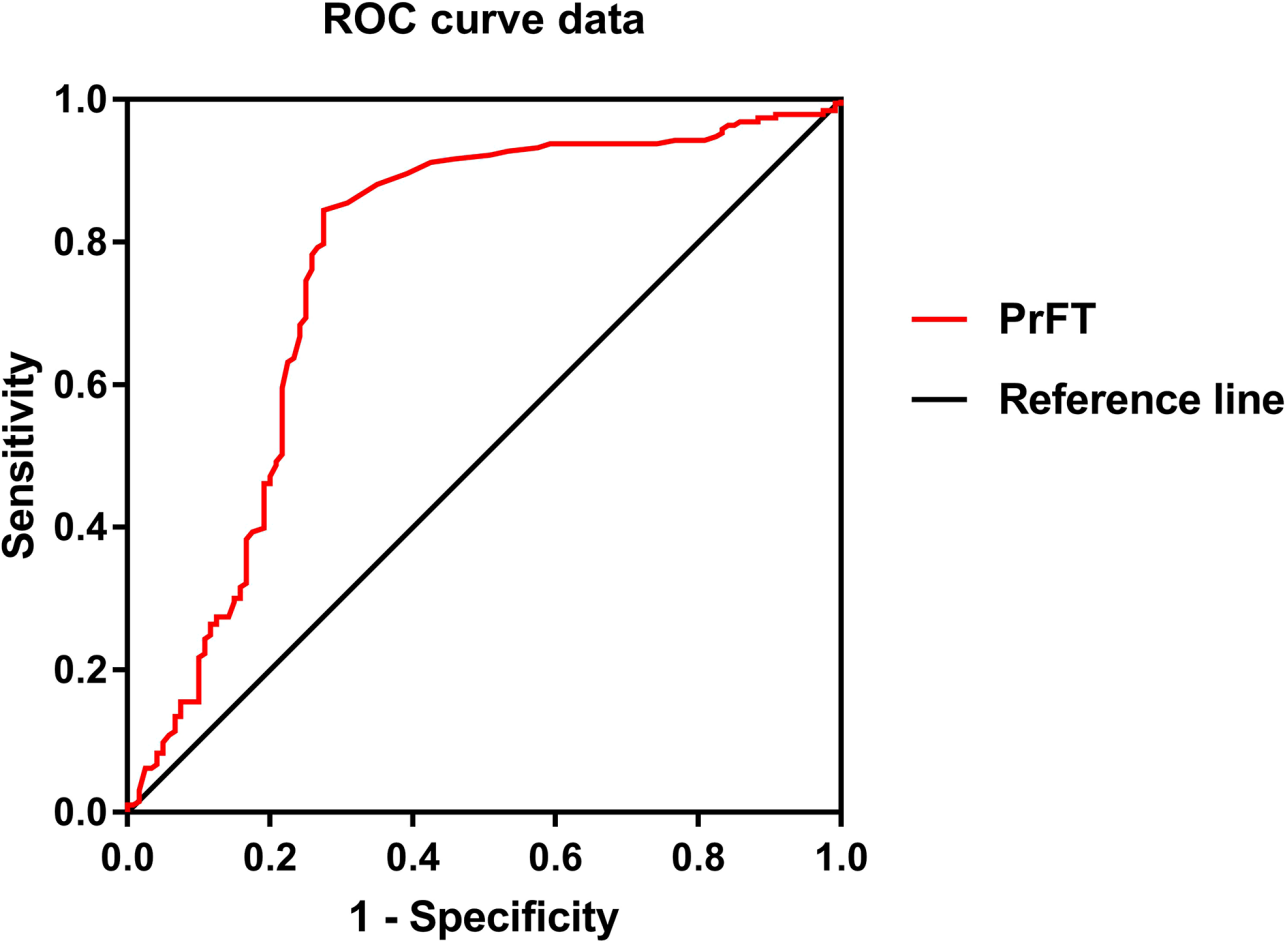
Receiver operating characteristic curves for the cutoff value of perirenal fat thickness (PrFT) to identify osteoporosis.

Binomial logistic regression analysis was also conducted to assess the independent variables of PrFT for osteoporosis. As shown in **Table 5**, PrFT was independently associated with osteoporosis after adjustment for age, diabetes duration, hypertension, sedentary behavior, smoking, and drinking (Model 4); the OR (95% CI) was 1.12 (1.05–1.20). A significant association between PrFT and osteoporosis was also found after additional adjustment for BMI, HbA1c, BMI, TG, HDL-c, LDL-c, creatinine, and UA (Model 5); the OR (95% CI) was 1.11 (1.04–1.20). In addition, the ORs remained significant after further adjustment for BTMs such as OC, β-CTX, 25-OH-D, iPTH, alkaline phosphatase (ALP), calcium, and phosphorus (Model 6); the OR (95% CI) was 1.13 (1.04–1.23). To determine the identifying value of PrFT for osteoporosis, ROC curve analysis was performed (**Figure 2**). The results showed that PrFT seems to have a relatively good identifying value for osteoporosis. The area under the curve (AUC) of PrFT in identifying osteoporosis was 0.766 (95% CI: 0.705–0.826, *P* < 0.001). The optimal cutoff value of PrFT was 15.2 mm (sensitivity: 72.5%, specificity: 79.8%).

**Table 5:** Binomial logistic regression analysis adjusted ORs (95% CIs) for the associations between PrFT and the risk of osteoporosis.

Models	B	Wald	OR(95%CI)	*P*
Model 4	0.117	11.67	1.12(1.05-1.20)	0.001
Model 5	0.107	8.25	1.11(1.04-1.20)	0.004
Model 6	0.126	8.93	1.13(1.04-1.23)	0.003

Model 4: Adjusted for age, diabetes duration, hypertension, sedentary behavior, smoking, and drinking. Model 5: Additional adjustment for BMI, HbA1c, TG, HDL-c, LDL-c, creatinine, and UA. Model 6: Additional adjustment for bone turnover markers such as OC, β-CTX, 25-OH-D, iPTH, alkaline phosphatase (ALP), calcium, and phosphorus based on Model 5.

PrFT, perirenal fat thickness; OR, odds ratio; BMI, body mass index; OC, osteocalcin; β-CTX, β-cross-linked C-telopeptide of type I collagen; 25-OH-D, 25-hydroxyvitamin D; iPTH, intact parathyroid hormone; HbA1c, glycated hemoglobin; UA, uric acid; TG, triglyceride; HDL-c, high-density lipoprotein cholesterol; LDL-c, low-density lipoprotein cholesterol.

## Discussion

Increasing evidence demonstrated that visceral fat can decrease BMD and compromise bone structure. Perirenal fat is a kind of measurable visceral fat; its role in bone metabolism still lacks evidence. In this cross-sectional study, we mainly aimed to assess the associations between PrFT and BTMs and BMD in postmenopausal women with T2DM and further explore the correlation between PrFT and osteoporosis. The most important findings of our study were that PrFT was negatively correlated with bone resorption markers β-CTX, L1–L4 BMD, and T-score. These correlations remained significant after adjustment for other confounding factors. Furthermore, PrFT was also independently associated with osteoporosis and showed a good identifying value for osteoporosis.

Obesity and T2DM have become major and growing public health problems that can increase fracture risk. Understanding how obesity and T2DM modulate fracture risk is important to identify and treat people at high risk of fracture. Most available evidence supported that obesity is positively associated with higher BMD through increasing mechanical loading and strain (18). Chen et al. (19) reported that changes in most fat tissue mass are more closely correlated with changes in regional BMD than lean tissue mass in healthy postmenopausal women. Unlike fat mass accumulated at the subcutaneous layer, visceral fat mass accumulation plays a harmful role in BMD by secreting pro-inflammatory cytokines such as IL-6 and TNF-α, which may increase bone resorption (9, 10). In support of these opposite effects of visceral fat on BMD, evidence from clinical studies showed that lower BMD and some adverse microstructural features were associated with greater central and visceral adiposity from bone biopsy and High resolution peripheral computed tomography parameters (HR-pQCT) (20–22). Among visceral adipose tissue deposits, perirenal fat is a kind of measurable visceral adipose tissue located in the retroperitoneal space and enclosed from the inner side of the abdominal musculature to the surface of the kidney. Anatomical studies confirmed that perirenal fat has a complete vascular supply and lymphatic system, which can provide the structural basis for perirenal fat regulating the cardiovascular, metabolism, and bone through neural reflexes, adipokine secretion, and adipocyte interactions (14, 15). Perirenal fat is highly active in adipokine synthesis that can directly influence bone homeostasis (14). Adiponectin is a kind of adipokine produced by adipose tissue and its receptor is expressed in osteoblasts, which may directly modulate bone metabolism. Several clinical studies observed that serum adiponectin is negatively associated with BMD and positively with bone formation and resorption markers (23, 24), which may indicate that adiponectin could be a negative regulator of bone mass. In contrast to clinical findings, most *in vitro* studies showed that adiponectin has an improved effect on osteoblasts and an inhibitory effect on osteoclasts, which may increase bone formation and decrease bone resorption (25, 26). Leptin is another kind of adipokine produced by white adipose tissue or bone marrow adipocytes that can regulate energy balance by signaling satiety in the hypothalamus and reduce food intake. More studies realized that leptin also plays a major role in bone metabolism; increased circulatory leptin levels can directly act on bone cells and increase bone formation (27); meanwhile, leptin can also inhibit bone formation by increasing the activation of the sympathetic nervous system when acting through the hypothalamus (28). In addition, perirenal fat is also active in cytokine synthesis by activating local immune cells. Simultaneously, the synthesis of pro-inflammatory cytokines (TNF-α) is enhanced in perirenal fat (29), which can suppress osteoblast activity at some stages of differentiation and stimulate osteoclast proliferation and differentiation (30). Based on the above studies, we can speculate that perirenal fat is involved in the development of bone homeostasis imbalance.

More insights have been put into how obesity influences bone metabolism by measuring bone turnover biochemical markers. Most studies that enrolled diabetic populations have consistent results that bone resorption marker β-CTX and bone formation marker OC were reduced (31), whereas these changes may vary with gender and age. Women in menopause have a rapid increase in bone turnover due to a rapid decrease in estrogen levels, which causes higher bone resorption and negative bone balance, leading to bone loss. Despite most studies having confirmed a negative association between VFA and OC and β-CTX in postmenopausal women (20, 32), fewer studies have explained the underlying relationships between perirenal fat and BTMs. A recent study that enrolled 234 T2DM patients reported that PrFT measured by ultrasound is negatively correlated with β-CTX (33). In the present study, the results are consistent with the above study; PrFT was negatively correlated with β-CTX after adjusting for potential confounding factors. Limited by the nature of our study and the above study that are cross-sectional studies, the exact association between PrFT and BTMs remains uncertain. Currently, most studies have found a negative association between VFA and BDM, whereas the relationship between perirenal fat and BMD remains uncertain. Cherif et al. (34) found a positive association between perirenal fat mass and *ex vivo* L1 BMD in overweight rats, while this correlation did not remain significant after adjusting for other confounding factors. Our results are consistent with previous studies reported in VFA; PrFT was negatively correlated with L1–L4 BMD (*r* = -0.351, *P><* 0.001), and this correlation remains significant after adjusting for potential confounding factors (*β* = -0.309, *P<* 0.001). In addition, PrFT was also independently associated with osteoporosis and seems to have a good identifying value for osteoporosis; more studies with enough follow-up should be conducted to further confirm these findings. Our study also found that participants in higher PrFT groups have higher levels of TG and UA and a lower level of HDL-c. These findings are consistent with those of other previous studies. Cross-sectional studies had observed that PrFT was associated with mean 24-h diastolic blood pressure (35), insulin resistance, HDL-c, and UA (36) in obese subjects.

To our knowledge, our study firstly put insight into the association between PrFT and bone metabolism in postmenopausal women with T2DM. The other strengths of this study adjusted for several potential confounding variables in the final analysis and included enough sample size that can represent the population of Chinese postmenopausal women with T2DM. Meanwhile, some limitations need to be mentioned. Firstly, our study was designed as a cross-sectional study without enough follow-up; it cannot directly illustrate the relationship between PrFT and bone metabolism. The underlying mechanisms were still unknown, and further studies are needed to evaluate the specific mechanisms. Secondly, the study population is composed of Chinese postmenopausal women with T2DM; our findings may not be applicable to other populations with different genders, ages, and races. Thirdly, we did not evaluate whether PrFT is a more powerful biomarker for BTMs or BMD rather than other indexes such as VFA.

In conclusion, this study observed that PrFT was negatively correlated with bone resorption markers β-CTX, L1–L4 BMD, and T-score. In addition, PrFT was also independently associated with osteoporosis and seems to have a relatively good identifying value for osteoporosis. These findings may indicate that perirenal fat may play an important role in bone metabolism, whereas more longitudinal studies are needed to further evaluate these findings and illustrate the underlying mechanisms.

## Data availability statement

The raw data supporting the conclusions of this article will be made available by the authors, without undue reservation.

## Ethics statement

The studies involving human participants were reviewed and approved by the Ethical Committee of Longyan First Affiliated Hospital of Fujian Medical University. The patients/participants provided their written informed consent to participate in this study.

## Author contributions

WW took charge of the software and contributed to writing— original draft. WW, RH, PT, MT, and XG conducted the investigation. XG contributed to data curation and writing-editing. All authors contributed to the article and approved the submitted version.

## Conflict of interest

The authors declare that the research was conducted in the absence of any commercial or financial relationships that could be construed as a potential conflict of interest.

## Publisher’s note

All claims expressed in this article are solely those of the authors and do not necessarily represent those of their affiliated organizations, or those of the publisher, the editors and the reviewers. Any product that may be evaluated in this article, or claim that may be made by its manufacturer, is not guaranteed or endorsed by the publisher.
